# Methods for selecting health concepts and domains from in-depth qualitative interviews for the development of patient reported outcome measures (PROMS): a case study

**DOI:** 10.1186/1745-6215-16-S2-O5

**Published:** 2015-11-16

**Authors:** Jane Blazeby, Leila Rooshenas, Rhiannon Macefield, Daisy Townsend, Tom Milne

**Affiliations:** 1 University of Bristol, Bristol, UK; 2 University of Birmingham, Birmingham, UK

## 

One fundamental step in PROM development is adequacy of item generation based on the conceptual framework of the instrument. When this framework is novel and not obvious, the US Federal Drug Administration (FDA) guidance for industry recommends cognitive patient interviews be undertaken to ensure understanding and completeness of the health domains contained in the questionnaire items. Documentation of the process by which items and domains are identified (often from patient interviews) is recommended; however, transparent methods are lacking. We propose novel and transparent methods for selecting PROM domains developed in a feasibility case study.

Inductive interviews were conducted with purposefully selected patients with experience of the clinical condition/intervention. An open-ended topic guide encouraged narratives of patients' experiences. Interviews were independently conducted and thematically analysed using the same electronic software package by two researchers, with an intention to code all aspects of patient experience. Codes were listed with supporting raw data (quotes), and discussed with a third independent researcher to agree an initial set of concepts (X identical codes, Y overlapping and Z new concepts only identified by one researcher). Further interviews tested the relevance and exhaustiveness of domains, with amendments noted and discussed. A final set of domains was incorporated into an initial PROM, supplemented with domains extracted from the literature and further patient interviews to establish face validity.

This is a transparent process and method for documenting PROM concept and domain selection from qualitative interviews through to questionnaire items. Reporting standards for this process are needed.

